# Association of Polymorphisms in Candidate Genes with the Litter Size in Two Sheep Breeds

**DOI:** 10.3390/ani9110958

**Published:** 2019-11-12

**Authors:** Zehu Yuan, Junxia Zhang, Wanhong Li, Weimin Wang, Fadi Li, Xiangpeng Yue

**Affiliations:** 1 State Key Laboratory of Grassland Agro-ecosystems, Key Laboratory of Grassland Livestock Industry Innovation, Ministry of Agriculture and Rural Affairs, Engineering Research Center of Grassland Industry, Ministry of Education, College of Pastoral Agriculture Science and Technology, Lanzhou University, Lanzhou 730020, China; yuanzh16@lzu.edu.cn (Z.Y.); limh@lzu.edu.cn (W.L.); lifd@lzu.edu.cn (F.L.); 2 College of Animal Science and Technology, Gansu Agricultural University, Lanzhou 730070, China; zhangjunxia999@sina.com (J.Z.); wangwm@gsau.edu.cn (W.W.); 3 College of Agriculture and Animal Husbandry, Qinghai University, Xi’ning 810016, China; 4 Engineering Laboratory of Sheep Breeding and Reproduction Biotechnology in Gansu Province, Minqin 733300, China

**Keywords:** MAS, SNP, litter size, sheep, SNPscan

## Abstract

**Simple Summary:**

Increasing litter size is critical for the intensive sheep production system. Genetic marker-assisted selection (MAS) based on proven molecular indicators could enhance the efficacy of sheep selection with improving litter size traits. Many single nucleotide polymorphisms (SNPs) linked with litter size in Hu sheep and Small-tailed Han sheep have been identified. However, they usually explain a small portion of genetic variation and additional genetic markers linked with litter size have not been found. The present study investigated the potential SNPs in ten genes as candidate markers for improved litter size in sheep. As a result, nine SNPs in six out of ten genes can be served as useful genetic markers to improve the selection of litter size since they are significantly associated with litter size either in Hu sheep or Small-tailed Han sheep. Further, a combined haplotypes analysis of the two loci (*LIFR*: g.35862868C>T and *LIFR*: g.35862947G>T) revealed that H2H3 (CTTT) combined haplotypes had the largest litter size than the rest combined haplotypes and more than those with either mutation alone in Small-tailed Han sheep. Our knowledge is essential to implement the MAS in sheep and further to increase the profitability in the sheep industry.

**Abstract:**

Hu sheep and Small-tailed Han sheep are the most widely raised and most famous maternal sheep breeds in China, which are known for precocious puberty, perennial oestrus and high fecundity (1–6 lambs each parity). Therefore, it is crucial to increase litter size of these two breeds for intensive sheep industry. The objective of this study was to identify potential genetic markers linked with sheep litter size located at ten genes. This study collected blood sample of 537 Hu sheep and 420 Small-tailed Han sheep with litter size of first parity. The average litter sizes in Hu sheep and Small-tailed Han sheep were 2.21 and 1.93. DNA-pooling sequencing method was used for detecting the potential single nucleotide polymorphisms (SNPs) in ten genes related to follicle development and female reproduction. SNPscan^®^ was used for individually genotyping. As a result, a total of 78 putative SNPs in nine out of ten candidate genes (except *NOG*) were identified. In total, 50 SNPs were successfully genotyped in Hu sheep and Small-tailed Han sheep. After quality control, a total of 42 SNPs in Hu sheep and 44 SNPs in Small-tailed Han sheep were finally used for further analysis. Association analysis revealed that nine SNPs within six genes (*KIT*: g.70199073A>G, *KITLG*: g.124520653G>C, *ADAMTS1*: g.127753565T>C, *ADAMTS1*: g.127754640G>T, *NCOA1*: g.31928165C>T, *NCOA1*: g.32140565G>A, *LIFR*: g.35862868C>T, *LIFR*: g.35862947G>T and *NGF*: g.91795933T>C) were significantly associated with litter size in Hu sheep or Small-tailed Han sheep. A combined haplotypes analysis of the two loci (*LIFR*: g.35862868C>T and *LIFR*: g.35862947G>T) revealed that H2H3 (CTTT) combined haplotypes had the largest litter size than the rest combined haplotypes and more than those with either mutation alone in Small-tailed Han sheep. Taken together, our study suggests that nine significant SNPs in six genes can be served as useful genetic markers for MAS in sheep.

## 1. Introduction

**Figure 1:**
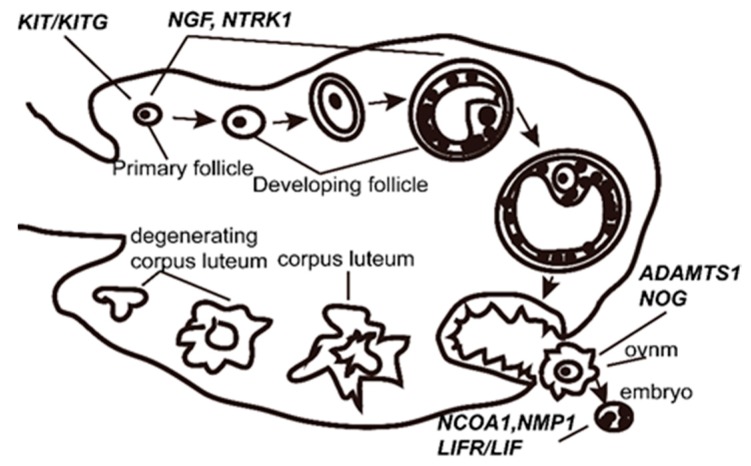
Reproductive physiology functions of the ten candidate genes.

Litter size is one of the most important economic traits because it has a noticeable impact on profitability in the sheep industry. If specific genetic makers associated with litter size are identified, marker-assisted selection (MAS) can be then used to improve the selection of litter size. Genomic selection is efficient but severely unaffordable in large sample size. Finding single nucleotide polymorphisms (SNPs) associated with litter size in candidate gene is an alternative method to improve the litter size in case of a limited budget. As well known, ovulation rate and embryo survival are directly linked to sheep litter size [1]. Therefore, it is an effective way to scan SNPs in genes with known reproductive physiology functions. Based on current literatures, ten genes (*NGF*, *NTRK1*, *KIT*, *KITLG*, *LIF*, *LIFR*, *NCOA1*, *ADAMTS1*, *NPM1,* and *NOG*) with known reproductive physiology functions (Figure 1, Supplementary Materials Table S1) in animals were selected as potential candidate genes for sheep litter size. Specifically, *KIT, KITG, NCF,* and *NTRK1* play roles in follicle growth [2,3,4,5,6,7,8]. Two single nucleotide polymorphisms (SNPs) in *KIT* were linked with goat litter size [9]. Similarly, ten loci in *KITLG* were significantly associated with goat litter size [10,11,12,13]. *ADAMTS1* and *NOG* were crucial for ovulation [14,15,16,17,18] and a mutation in *ADAMTS1* was notably linked with litter size in a new Qingping female line [19]. *NCOA1* was essential for embryo survival [20]. A significant association of SNPs in the *NCOA1* gene with litter size in Icelandic sheep has been reported [21]. *LIFR*, *LIF* and *NMP1* [22,23,24,25,26,27,28] play a vital role in embryo implantation. *LIF* linked with litter size in pigs has been reported [29].

In China, the population size of domesticated sheep was 116.35 million in 2017 and sheep meat accounts for 14.38 % for the meat production in the ruminant sector (http://www.fao.org/faostat/, last access date: 31 October 2019). Hu sheep and Small-tailed Han sheep are two majors, widely used high-fecundity sheep breeds for intensive sheep production system (house feeding) in China. The estimated inventory of Hu sheep and Small-tailed Han sheep were approximately four million and five million, respectively. These two breeds usually used as female parents crossing with elite ram breeds, e.g., polled Dorset, to produce meat.

Despite the relatively clear characterization of the roles of these ten genes in influencing the reproductive biological process, the assessment of these candidate genes for the genetic selection of sheep litter size has not been conducted extensively and systematically, particularly in Hu sheep and Small-tailed Han sheep. Our hypothesis for this study was that the potential SNPs in above mentioned ten candidate genes may be linked with litter size in Hu sheep and Small-tailed Han sheep. Thus, the objectives of this study were to scan SNPs in these ten genes and to investigate the association of SNPs with litter size in Hu sheep (n = 537) and Small-tailed Han sheep (n = 420). Our findings may serve as useful genetic markers for Hu sheep and Small-tailed Han sheep.

## 2. Materials and Methods 

The experimental protocols followed the guidelines stated in the Guide for the Use of Animal Subjects in Lanzhou University and the Rules and Regulations of Experimental Field Management Protocols (file No: 2010-1 and 2010-2), which were approved by Lanzhou University.

### 2.1. Phenotypic Data Collection and DNA Extraction

**Figure 2:**
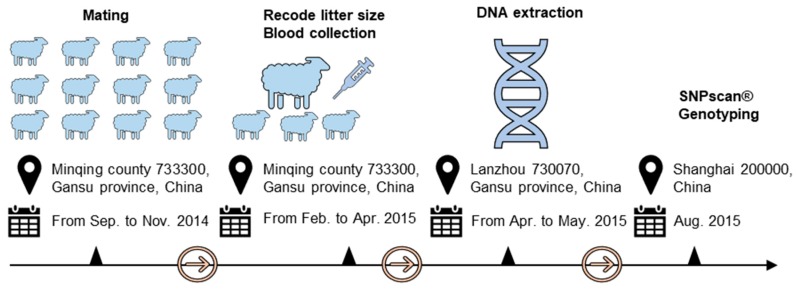
Schematic diagram of the experimental protocol across times.

**Figure 3:**
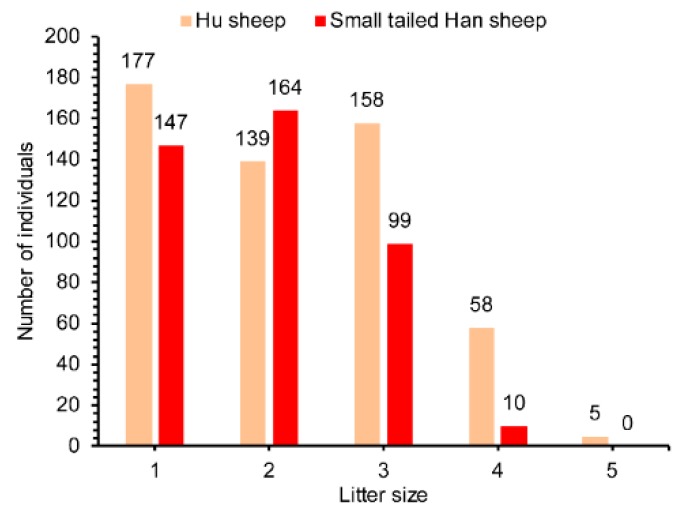
Frequency distribution of litter size in Hu sheep and Small-tailed Han sheep.

Increasing litter size is critical for intensive sheep production system (house feeding). Two sheep breeds, Hu sheep and Small-tailed Hans sheep were selected in this study because these two breeds are widely used in China for their high fecundity. In the current study, all ewes (Hu sheep and Small-tailed Han sheep) were raised under the same management conditions and were mated from September to December 2014, which was a short-day period in Minqing, Gansu province, China. Blood samples of 957 ewes with litter size recorded at first parity from February to April 2015 were collected at Minqing Zhongtian Sheep Industry Co., Ltd. (Minqing, Gansu Province, P.R. China), including 537 Hu sheep and 420 Small-tailed Han sheep. The experimental protocol across times was shown in Figure 2. Data for the phenotype of litter size records in Hu sheep and Small-tailed Han sheep are shown in Figure 3. The litter size ranged from one to five in Hu sheep and one to four in Small-tailed Han sheep. The average litter sizes in Hu sheep and Small-tailed Han sheep were 2.21 and 1.93. Genomic DNA was extracted using a phenol-chloroform method [30] and then dissolved in double-distilled water and stored at −20 °C.

### 2.2. Single Nucleotide Polymorphism Detection and Genotyping

A total of ten DNA pools were constructed for two sheep breeds (each five) to identify potential SNPs in the ten genes analyzed. Each DNA pool comprised ten individuals which were randomly selected balanced across all different litter sizes, as described in Table 1. Different DNA pools contain the different individuals. A total of 119 paired primers were designed to amplify all exons and flanking regions of each gene (Supplementary Materials Table S1). The PCR reaction mixture was 25 μL, containing 1 μL pooled DNA, 0.4 μL forward primers, 0.4 μL reverse primers, 12.5 μL 2× Taq PCR MasterMix and 10.7 μL double-distilled water. The PCR protocol was 5 min at 94 °C for initial denaturing followed by 35 cycles at 94°C for 30 s, annealing for 30 s, 72°C for 30 s, and a final extension at 72°C for 5 min. The purified PCR products were sequenced using an ABI 3730XL DNA analyzer (Applied Biosystems, Foster City, CA), and the sequences were compared using DNAstar to detect putative SNPs. The identified SNPs were individually genotyped by the SNPscan^®^ method (Genesky Biotechnologies Inc., Shanghai, China), which was based on double ligation and multiplex fluorescence PCR [31]. Three DNA samples were randomly selected from 957 samples to genotype twice, and two blank samples (double-distilled water) were also used to eliminate cross-contamination.

### 2.3. Population Parameter Calculation and Quality Control

The SnpReady R package [32] was used to calculate the allele frequencies, minor allele frequency (MAF), polymorphic information content (PIC), expected heterozygosity (He), and observed heterozygosity (Ho) and to implement the Hardy–Weinberg equilibrium test. The formulas used to estimate population parameters were well descripted in the manual of SnpReady R package [32]. SNP was excluded for further analysis if its MAF was smaller than 0.05 and/or it seriously deviated from Hardy–Weinberg equilibrium (*p* < 0.001).

### 2.4. Single Nucleotide Polymorphism Association Analysis

A linear model was used to test the single SNP effect on the litter size in each sheep breed:

**L** = **1**_***n***_ μ + **G** + **e** (1)

where **L** was an n × 1 vector of the litter size (Hu sheep, n = 537; Small-tailed Han sheep, n = 420), **1*_n_*** was an n × 1 vector with all elements equal to 1, μ was the overall mean, **G** was fixed effects corresponding to SNPs, and **e** was an n × 1 vector of random residual effect. Differences in mean litter size among genotypes were tested using the LSD test in the agricolae R package [33]. *P* < 0.05 was to be considered significant. *P* < 0.01 was considered to be highly significant. Bonferroni correction was applied for multiple testing between genotype groups. 

### 2.5. The Haplotype Analysis

In the current study, only the significant SNPs in the same gene in each sheep breed were used to estimate the extent of linkage disequilibrium (LD) and haplotype blocks. Two significant SNPs (*ADAMTS*1: g.127753565T>C and *ADAMTS1*: g.127754640T>G) in Hu sheep and four significant SNPs (*NCOA1*: g.31928165C>T, *NCOA1*: g.32140565G>A, *LIFR*: g.35862868C>T and *LIFR*: g.35862947G>T) in Small-tailed Han sheep meet this precondition and were used for further analysis. The extent of LD between significant SNP pairs and haplotype blocks was estimated using the Haploview 4.2 software [34]. The model of association analysis between combined haplotypes and litter size was as follows in Formula (1) except that the genotype (G) was replaced by combined haplotypes. Numbers less than 12 of the combined haplotypes were excluded from the association analysis.

## 3. Results

### 3.1. Genotyping and Quality Control

A total of 78 putative SNPs in nine candidate genes (except *NOG*) were identified by DNA pooling sequencing (Supplementary Materials Table S2). After evaluating the ligand primers, 50 out of 78 SNPs were genotyped using the SNPscan^®^ method (Supplementary Table S2). The genotypic correlation coefficient of three pairs of technical repeats equal to one and no genotyping signals were detected in two blank samples, indicating that the SNPscan^®^ method is reliable. Eight SNPs (*KIT*: g.70224398T>A, *ADAMTS1*: g.127751615C>T, *ADAMTS1*: g.127753643C>T, *ADAMTS1*: g.127756130G>A, *NPM1*: g.3247135T>G, *NPM1*: g.3247450C>T*, ADAMTS1*: g.127753727C>T; and *LIF*: g.68801067C>T) in Hu sheep and six SNPs in Small-tailed Han sheep (*KIT*: g.70224398T>A, *ADAMTS1*: g.127756130G>A, *NPM1*: g.3247135T>G, *NPM1*: g.3247450C>T, *ADAMTS1*: g.127753727C>T, and *LIFR*: g.35867028T>C) were excluded for further analysis because their MAF were smaller than 0.05 and/or they seriously deviated from Hardy–Weinberg equilibrium (*p* < 0.001). Finally, a total of 42 SNPs in Hu sheep and 44 SNPs in Small-tailed Han sheep were used for further analysis (Table 2).

### 3.2. Population Genetic Parameters

The MAF, He, Ho, PIC and Hardy–Weinberg equilibrium test *p*-values are summarized in Table 2. The MAF in Hu sheep ranged from 0.06 to 0.48, and that in Small-tailed Han sheep ranged from 0.11 to 0.49. The minimum (maximum) values of He were 0.12 (0.5) and 0.19 (0.5) in Hu sheep and Small-tailed Han sheep, respectively. The min (max) value of Ho was 0.12 (0.37) and 0.19 (0.56) in Hu sheep and Small-tailed Han sheep. Nine SNPs in Hu sheep (*KITLG*: g.124520653G>C, *NCOA1*: g.31928230C>T, *NCOA1*: g.32140565G>A, *LIF*: g.68816215C>T, *LIFR*: g.35817147A>G, *LIFR*: g.35817247G>A, *LIFR*: g.35835474G>T, *LIFR*: g.35841608T>C, and *LIFR*: g.35847837A>G) and seven SNPs in Small-tailed Han sheep (*KITLG*: g.124520653G>C, *ADAMTS1*: g.127751615C>T, *LIF*: g.68801067C>T, *LIF*: g.68816215C>T, *LIFR*: g.35845633T>C, *LIFR*: g.35853852T>C, and *NTRK1*: g.105276945C>T) with low polymorphic status (PIC < 0.25) were observed. The other SNPs had moderate (0.25 ≤ PIC < 0.5) or high polymorphic (PIC ≥ 0.5) status. One SNP (*NTRK1*: g.105288550C>G) in Hu sheep and nine SNPs (*KIT*: g.70199073A>G, *NPM1*: g.3246266T>G, *LIFR*: g.35813931G>A, *LIFR*: g.35813935A>G, *LIFR*: g.35847864A>T, *LIFR*: g.35848108A>G, *LIFR*: g.35862868C>T, *LIFR*: g.35862947G>T, and *NTRK1*: g.105288550C>G) in Small-tailed Han sheep deviated from Hardy–Weinberg equilibrium (*p* < 0.05).

### 3.3. Single Nucleotide Polymorphisms Associated with Litter Size

Table 3 details the significant associations between the tested SNPs and litter size. Single SNP association analysis showed that three SNPs (*KITLG*: g.124520653G>C, *ADAMTS1*: g.127754640T>G, and *ADAMTS1*: g.127753565T>C) were significantly associated with litter size in Hu sheep, four SNPs (*NCOA1*: g.31928165C>T, *NCOA1*: g.32140565G>A, *LIFR*: g.35862947G>T, and *NGF*: g.91795933T>C) were significantly associated with litter size in Small-tailed Han sheep, two SNPs (*KIT*: g.70199073A>G and *LIFR*: g.35862868C>T) were strongly associated with litter size in Small-tailed Han sheep. *LIFR*: g.35862868C>T was the most significant locus in Small-tailed Han sheep (*p* = 0.0054). In this locus, sheep with the TT and CT genotypes were significantly greater than CC genotype carriers on litter size, which could be observed with overall litters size 0.33 and 0.29 greater than CC genotype carriers, respectively. Overall, nine SNPs (*KIT*: g.70199073A>G, *KITLG*: g.124520653G>C, *ADAMTS1*: g.127753565T>C, *ADAMTS1*: g.127754640T>G, *NCOA1*: g.31928165C>T, *NCOA1*: g.32140565G>A, *LIFR*: g.35862868C>T, *LIFR*: g.35862947G>T, and *NGF*: g.91795933T>C) were significantly associated with litter size in at least one sheep population (Table 3). The remaining 36 SNPs were not significantly associated with litter size in any population (Supplementary Materials Table S3).

Nine significant SNPs were mapped to sheepQTL database (https://www.animalgenome.org/cgi-bin/QTLdb/OA/index) to better understand the potential role of these SNPs. Nine SNPs were mapped to 13 litter size related quantitative trait loci (QTLs) (Table 4). The nearest distance between SNPs and QTLs is 1.8 Mb (between *KIT*: g.70199073A>G and QTL 13975). 

### 3.4. The Haplotype Analysis

**Figure 4:**
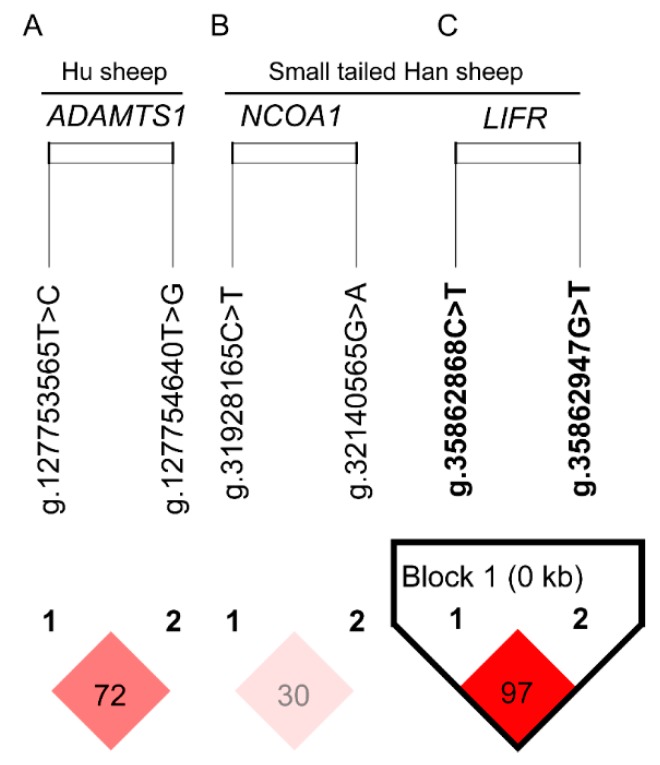
Linkage disequilibrium pattern for the significant single nucleotide polymorphisms (SNPs) in *ADAMTS1* (A), *NCOA1* (B), and *LIFR* gene (C). Scale of red color indicating the extend linkage disequilibrium (D′ value).

**Figure 5:**
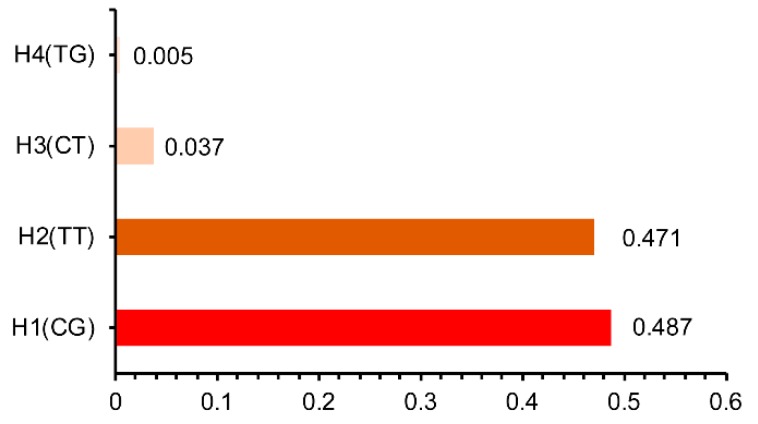
Frequency of haplotypes inferred form two loci (*LIFR*: g.35862868C>T and *LIFR*: g.35862947G>T) in Small-tailed Han sheep.

The D′ (r^2^) value is a factional indicator of LD and was calculated in this study. The D′ (r2) were 0.72 (0.34) and 0.30 (0.04) indicated that two SNPs (*ADAMTS1*: g.127753565T>C and *ADAMTS1*: g.127754640T>G) in Hu sheep (Figure 4A) and two SNPs (*NCOA1*: g.31928165C>T and *NCOA1*: g.32140565G>A) in Small-tailed Han sheep (Figure 4B) were not closely linked. While two SNPs *(LIFR*: g.35862868C>T and *LIFR*: g.35862947G>T) were closely linked (D′ and r2 were 0.97 and 0.84, Figure 4C). One haplotype block was identified in Small-tailed Han sheep (Figure 4C), where four different haplotypes were H1 (CG), H2 (TT), H3 (CT) and H4 (TG) (Figure 5). The highest haplotype frequency was H1, which accounted for 48.7% of all haplotypes. H2 was the next largest proportion, at 47.1% followed closely by H3 (3.7%) and H4 (0.5%) (Figure 4). Since haplotypes with low frequencies were meaningless in statistical analysis, H4 was excluded from subsequent analysis. The association analysis showed that haplotype combinations were significantly associated with litter size in Small-tailed Han sheep (Table 5). Individuals with H2H3 (CTTT), H2H2 (TTTT), H1H2 (CTGT) had greater litter size than individuals with H1H1 (CCGG).

## 4. Discussion

Our hypothesis for this study was that the potential SNPs in these ten candidate genes may be linked with litter size in Hu sheep and Small-tailed Han sheep. If SNPs from the candidate genes were found significantly associated with litter size in a sheep breed, they could be served as molecular indicators for MAS. MAS based on prioritized molecular indicators could enhance the efficacy of sheep selection with improved litter size traits. 

Nine SNPs in Hu sheep and eight SNPs in Small-tailed Han sheep with low polymorphic status (PIC < 0.25) were observed, and the heterozygosity of these loci were also notably low, which might be a result of artificial selection [38]. The majority of sites had a moderate (0.25 ≤ PIC < 0.5) or high polymorphic status (PIC ≥ 0.5), suggesting that these SNPs could provide more effective genetic information. One SNP in Hu sheep and nine SNPs in Small-tailed Han sheep significantly deviated from Hardy–Weinberg equilibrium, which was mainly due to nonrandom mating and/or artificial selection [38].

In the current study, nine SNPs in six genes were strongly linked with sheep litter size, confirming their genetic roles in regulating sheep litter size. Previous research found two SNPs in *KIT* [9] and ten SNPs in *KITLG* were significantly associated with goat litter size [10,11,12,13]. A mutation in *ADAMTS1* was associated with pig litter size [19]. SNPs in *NCOA1* were associated with litter size in Icelandic sheep [21]. Two SNPs in *NGF* were associated with litter size in goat [39]. In accordance with these studies, seven SNPs (*KIT*: g.70199073A>G, *KITLG*: g.124520653G>C, *ADAMTS1*: g.127753565T>C, *ADAMTS1*: g.127754640G>T; *NCOA1*: g.31928165C>T, *NCOA1*: g.32140565G>A, and *NGF*: g.91795933T>C) in five genes were significantly associated with litter size in Hu sheep or Small-tailed Han sheep. To the best of our knowledge, this report is the first to describe two SNPs (*LIFR*: g.35862868C>T, *LIFR*: g.35862947G>T) in *LIFR* that were significantly associated with sheep litter size. As a result, the finding that nine genetic markers were significantly associated with litter size in Hu sheep or Small-tailed Han sheep in the present study suggests that these SNPs were in LD with a nearby QTL for litter size or directly involved in the genetic control of the trait. To date, four QTLs on chromosome six, one QTL on chromosome three, three QTLs on chromosome 16 have been reported to contribute to the litter size (total lambs born) in sheep (Table 4). In this study, nine significant SNPs were not exactly located in any of these sheep litter size QTLs, the relatively short distances between SNPs and QTLs (e.g., 1.8 Mb between *KITLG*: g.124520653G>C and QTL 13975) indicated that it is possible that these markers are in LD with sheep litter size QTLs.

Previous research focused mainly on missense mutations because these SNPs can change amino acids and then protein function directly. Many missense SNPs, such as Y409N in *KIT* [9], have been identified as litter size-related genetic markers in sheep [40] or other mammals [9]. In the current study, eight out of nine significant SNPs (*KIT*: g.70199073A>G; *KITLG*: g.124520653G>C, *ADAMTS1*: g.127754640G>T; *NCOA1*: g.31928165C>T; *NCOA1*: g.32140565G>A; *LIFR*: g.35862868C>T; *LIFR*: g.35862947G>T; and *NGF*: g.91795933T>C) are in the intronic region, and one SNP (*ADAMTS1*: g.127753565T>C) is a synonymous mutation (Ser→Ser), indicating that these SNPs are unlikely to affect litter size by changing the amino acid directly. Nevertheless, the effects of these mutations cannot be ignored because mutations in an intron can regulate gene expression [41] or splicing [42], and synonymous mutations can affect mRNA splicing [43], stability [44,45], protein translation, and folding [46]. Recent research has shown that a SNP (*KITLG*: c.1457A>C) in *KITLG* 3′-UTR can affect goat litter size by changing the microRNA binding site to regulate *KITLG* mRNA expression level [13]. This finding suggests that these mutations have potential functions in litter size, although the underlying genetic mechanism warrants further investigation.

It is necessary to analyze the combined effect of multiple loci on litter size, since the litter size is controlled by multiple genes and loci. In this research, associations between the combined haplotypes (in *LIFR* gene) and litter size of Small-tailed Han sheep were analyzed. The combined haplotypes of the two loci association analysis revealed that H2H3 (CTTT) combined haplotypes had larger litter sizes than other combined haplotypes and larger than those with either mutation alone in Small-tailed Han sheep. This result indicates that the *LIFR*: g.35862868C>T and *LIFR*: g.35862947G>T have an interaction effect on sheep litter size. A previous study showed that a combined genotype (TTAA) in *KIT* gene in three goat breeds showed a larger litter size than those carrying single mutations, which supported the interactive effect of multiple markers within a gene on the litter size [9]. Theoretically, the most positive combined haplotypes should be H2H2 (TTTT) based on the association analysis between single locus and litter size. However, the litter size between H2H2 and H2H3 was not significant, which suggests that both CTTT and TTTT were advantageous combinations affecting the sheep litter size.

We observed that parity had an important effect on sheep litter sizes because it was reported that the correlation between litter size and first to third parities was a significant positive correlation [40]. However, in the current study, only first parity data were collected. Thus, the model of the association analysis did not consider parities for the ewes, which would be essential for future studies.

## 5. Conclusions

In conclusion, we extensively and systematically investigated potential SNPs in ten genes (*NGF*, *NTRK1*, *KIT*, *KITLG*, *LIF*, *LIFR*, *NCOA1*, *ADAMTS1*, *NPM1,* and *NOG*) as candidate markers for improved litter size in sheep. Nine SNPs in six genes (*KIT*: g.70199073A>G, *KITLG*: g.124520653G>C, *ADAMTS1*: g.127753565T>C, *ADAMTS1*: g.127754640G>T, *NCOA1*: g.31928165C>T, *NCOA1*: g.32140565G>A, *LIFR*: g.35862868C>T, *LIFR*: g.35862947G>T, and *NGF*: g.91795933T>C) were significantly associated with litter size in Hu sheep or Small-tailed Han sheep. The combined haplotypes analysis of the two loci (*LIFR*: g.35862868C>T and *LIFR*: g.35862947G>T) revealed that H2H3 (CTTT) combined haplotypes had more litter size than other combined haplotypes and more than those with either mutation alone in Small-tailed Han sheep. These nine SNPs can be served as useful genetic markers for MAS in Small-tailed Han sheep and Hu sheep.

## Figures and Tables

**Table 1:** Sample constitution in each DNA pool balanced across different litter sizes.

Sheep Breed	Litter Size					Total Samples
	1	2	3	4	5	
Hu sheep (sample size)	3	2	2	2	1	10
Small-tailed Han sheep (sample size)	3	3	2	2	0	10

**Table 2:** Population genetic parameters of the 42 single nucleotide polymorphisms (SNPs) in Hu sheep (HUS) and 44 SNPs in Small-tailed Han sheep (STH).

SNP ID	Breed	MA	MAF	He	Ho	PIC	χ^2^	*p* Value
*KIT*: g.70199073A>G	HUS	G	0.19	0.30	0.31	0.26	0.21	0.6442
	STH	G	0.28	0.40	0.44	0.32	4.10	0.0430
*KITLG*: g.124520653G>C	HUS	C	0.10	0.19	0.19	0.17	0.01	0.9410
	STH	C	0.12	0.22	0.23	0.19	1.31	0.2530
*ADAMTS1*: g.127751615C>T	HUS	-	-	-	-	-	-	-
	STH	T	0.11	0.20	0.19	0.18	0.18	0.6729
*ADAMTS1*: g.127753565T>C	HUS	C	0.37	0.47	0.50	0.36	2.05	0.1519
	STH	C	0.46	0.50	0.53	0.37	1.93	0.1652
*ADAMTS1*: g.127753643C>T	HUS	-	-	-	-	-	-	-
	STH	T	0.20	0.32	0.31	0.27	0.45	0.5023
*ADAMTS1*: g.127754640T>G	HUS	T	0.48	0.50	0.54	0.37	3.19	0.0740
	STH	G	0.45	0.50	0.49	0.37	0.01	0.9216
*NCOA1*: g.31928165C>T	HUS	T	0.40	0.48	0.51	0.36	2.89	0.0891
	STH	T	0.33	0.44	0.45	0.35	0.10	0.7507
*NCOA1*: g.31928230C>T	HUS	T	0.15	0.26	0.27	0.23	0.83	0.3630
	STH	T	0.25	0.38	0.35	0.31	1.71	0.1916
*NCOA1*: g.32072394C>T	HUS	T	0.19	0.31	0.30	0.26	0.92	0.3371
	STH	T	0.22	0.34	0.36	0.28	1.54	0.2142
*NCOA1*: g.32116034A>G	HUS	G	0.22	0.34	0.32	0.28	1.60	0.2064
	STH	G	0.20	0.32	0.33	0.27	0.75	0.3867
*NCOA1*: g.32140565G>A	HUS	A	0.11	0.20	0.22	0.18	2.84	0.0922
	STH	A	0.20	0.32	0.32	0.27	0.03	0.8540
*NCOA1*: g.32140837T>C	HUS	C	0.24	0.36	0.34	0.30	2.13	0.1448
	STH	C	0.24	0.36	0.38	0.29	1.28	0.2589
*NPM1*: g.3245714T>C	HUS	C	0.43	0.49	0.49	0.37	0.04	0.8432
	STH	T	0.45	0.50	0.46	0.37	2.68	0.1019
*NPM1*: g.3245741C>T	HUS	T	0.43	0.49	0.50	0.37	0.07	0.7901
	STH	C	0.46	0.50	0.47	0.37	0.91	0.3407
*NPM1*: g.3245965C>T	HUS	T	0.43	0.49	0.49	0.37	0.01	0.9257
	STH	C	0.45	0.49	0.45	0.37	3.05	0.0808
*NPM1*: g.3246266T>G	HUS	G	0.24	0.36	0.34	0.30	3.12	0.0774
	STH	G	0.20	0.32	0.28	0.27	6.62	0.0101
*NPM1*: g.3247499A>T	HUS	T	0.29	0.41	0.44	0.33	2.04	0.1534
	STH	T	0.31	0.43	0.41	0.34	0.65	0.4218
*NPM1*: g.3251189A>T	HUS	T	0.42	0.49	0.49	0.37	0.00	0.9940
	STH	T	0.46	0.50	0.48	0.37	0.77	0.3811
*LIF*: g.68801067C>T	HUS	-	-	-	-	-	-	-
	STH	T	0.12	0.21	0.20	0.19	1.63	0.2019
*LIF*: g.68816215C>T	HUS	T	0.06	0.12	0.12	0.11	0.59	0.4418
	STH	T	0.11	0.19	0.19	0.17	0.00	0.9715
*LIFR*: g.35813711C>T	HUS	T	0.43	0.49	0.50	0.37	0.33	0.5646
	STH	T	0.32	0.43	0.44	0.34	0.03	0.8658
*LIFR*: g.35814094C>T	HUS	T	0.43	0.49	0.51	0.37	0.59	0.4418
	STH	T	0.32	0.44	0.44	0.34	0.10	0.7568
*LIFR*: g.35813931G>A	HUS	T	0.19	0.31	0.28	0.26	3.33	0.0680
	STH	T	0.29	0.41	0.37	0.33	3.88	0.0489
*LIFR*: g.35813935A>G	HUS	G	0.45	0.50	0.47	0.37	1.22	0.2690
	STH	G	0.35	0.46	0.41	0.35	4.52	0.0334
*LIFR*: g.35817147A>G	HUS	G	0.17	0.28	0.27	0.24	0.97	0.3250
	STH	G	0.27	0.40	0.40	0.32	0.00	0.9578
*LIFR*: g.35817247G>A	HUS	A	0.17	0.28	0.27	0.24	0.97	0.3250
	STH	A	0.27	0.40	0.40	0.32	0.00	0.9888
*LIFR*: g.35835329G>A	HUS	A	0.39	0.48	0.46	0.36	0.40	0.5280
	STH	A	0.43	0.49	0.53	0.37	2.54	0.1113
*LIFR*: g.35835474G>T	HUS	T	0.07	0.13	0.13	0.12	0.14	0.7117
	STH	T	0.23	0.35	0.37	0.29	0.66	0.4154
*LIFR*: g.35841608T>C	HUS	C	0.09	0.16	0.16	0.14	0.27	0.6044
	STH	C	0.23	0.35	0.37	0.29	1.07	0.3022
*LIFR*: g.35845633T>C	HUS	T	0.24	0.36	0.35	0.30	0.79	0.3743
	STH	C	0.15	0.25	0.27	0.22	1.64	0.2009
*LIFR*: g.35847837A>G	HUS	G	0.09	0.17	0.18	0.15	0.72	0.3954
	STH	G	0.25	0.37	0.38	0.30	0.43	0.5103
*LIFR*: g.35847864A>T	HUS	T	0.41	0.48	0.50	0.37	0.89	0.3463
	STH	T	0.42	0.49	0.54	0.37	5.34	0.0209
*LIFR*: g.35848079G>A	HUS	A	0.38	0.47	0.48	0.36	0.13	0.7151
	STH	A	0.42	0.49	0.52	0.37	2.27	0.1318
*LIFR*: g.35848108A>G	HUS	G	0.41	0.48	0.51	0.37	1.28	0.2571
	STH	G	0.45	0.49	0.56	0.37	8.43	0.0037
*LIFR*: g.35847912C>T	HUS	T	0.41	0.48	0.49	0.37	0.03	0.8553
	STH	T	0.43	0.49	0.53	0.37	2.63	0.1047
*LIFR*: g.35851829T>C	HUS	C	0.22	0.34	0.34	0.28	0.00	0.9701
	STH	C	0.18	0.30	0.30	0.25	0.33	0.5640
*LIFR*: g.35853589T>C	HUS	C	0.35	0.45	0.46	0.35	0.04	0.8470
	STH	C	0.42	0.49	0.51	0.37	0.70	0.4014
*LIFR*: g.35853637T>G	HUS	G	0.30	0.42	0.41	0.33	0.17	0.6765
	STH	G	0.37	0.46	0.50	0.36	2.46	0.1167
*LIFR*: g.35853852T>C	HUS	C	0.22	0.34	0.32	0.28	0.98	0.3211
	STH	C	0.13	0.23	0.23	0.20	0.01	0.9251
*LIFR*: g.35862868C>T	HUS	T	0.44	0.49	0.49	0.37	0.06	0.8061
	STH	T	0.48	0.50	0.55	0.37	4.63	0.0314
*LIFR*: g.35862947G>T	HUS	T	0.45	0.49	0.49	0.37	0.09	0.7616
	STH	G	0.49	0.50	0.55	0.37	5.07	0.0244
*LIFR*: g.35867028T>C	HUS	C	0.42	0.49	0.48	0.37	0.08	0.7815
	STH	-	-	-	-	-	-	-
*NGF*: g.91795933T>C	HUS	C	0.47	0.50	0.52	0.37	0.79	0.3734
	STH	C	0.40	0.48	0.46	0.37	0.72	0.3961
*NTRK1*: g.105276945C>T	HUS	T	0.34	0.45	0.42	0.35	3.18	0.0745
	STH	T	0.17	0.28	0.31	0.24	3.05	0.0807
*NTRK1*: g.105288550C>G	HUS	C	0.47	0.50	0.44	0.37	6.71	0.0096
	STH	C	0.46	0.50	0.44	0.37	5.47	0.0193

Note: MA: Minor allele; MAF: Minor allele frequency; PIC: Polymorphic information content; He: Expected heterozygosity; Ho: Observed heterozygosity; **χ^2^** and *p* value denote the chi-square value and *p*-value of Hardy–Weinberg equilibrium test; “-” denotes information is not available.

**Table 3:** Associations between the nine single nucleotide polymorphisms (SNPs) and litter size in Hu sheep and Small-tailed Han sheep.

SNP ID	Gene Symbol	Genotype	Hu Sheep		Small-Tailed Han Sheep	
			N	Mean ± S.D.	N	Mean ± S.D.
*KIT*: g.70199073A>G	*KIT* (intron 2)	AA	354	2.25 ± 1.05	211	1.88 ± 0.78b
		GA	166	2.13 ± 1.02	185	1.94 ± 0.85ab
		GG	17	2.12 ± 1.32	24	2.42 ± 0.83a
		*p*-value	0.42		0.00945 **	
*KITLG*: g.124520653G>C	*KITLG* (intron 9)	CC	6	3.17 ± 0.75a	4	1.50 ± 0.58
		GC	100	2.10 ± 1.11b	97	1.93 ± 0.79
		GG	431	2.22 ± 1.03ab	319	1.94 ± 0.84
		*p*-value	0.0461 *		0.568	
*ADAMTS1*: g.127753565T>C	*ADAMTS1* (exon 5) Ser→Ser	CC	66	2.24 ± 1.02ab	114	2 ± 0.79
		CT	266	2.09 ± 1.02b	223	1.93 ± 0.85
		TT	205	2.35 ± 1.07a	83	1.84 ± 0.80
		*p*-value	0.0262 *		0.42	
*ADAMTS1*: g.127754640G>T	*ADAMTS1* (intron 7)	GG	137	2.39 ± 1.07a	86	1.88 ± 0.79
		GT	288	2.10 ± 1.01b	207	1.94 ± 0.83
		TT	111	2.25 ± 1.11ab	127	1.95 ± 0.84
		*p*-value	0.0297*		0.817	
*NCOA1*: g.31928165C>T	*NCOA1* (intron 1)	CC	184	2.28 ± 1.09	185	1.98 ± 0.86a
		CT	276	2.13 ± 1.01	189	1.97 ± 0.79a
		TT	76	2.3 ± 1.08	45	1.60 ± 0.75b
		*p*-value	0.217		0.0158*	
*NCOA1*: g.32140565G>A	*NCOA1* (intron 21)	AA	3	3.00 ± 1.70	16	1.38 ± 0.50b
		GA	116	2.25 ± 1.02	135	2.02 ± 0.84a
		GG	418	2.19 ± 1.05	269	1.92 ± 0.82a
		*p*-value	0.368		0.0109*	
*LIFR*: g.35862868C>T	*LIFR* (intron 19)	CC	169	2.19 ± 1.03	104	1.71 ± 0.81b
		CT	262	2.21 ± 1.08	231	2.00 ± 0.83 a
		TT	106	2.25 ± 1.01	84	2.04 ± 0.78a
		*p*-value	0.91			0.0054**
*LIFR*: g.35862947G>T	*LIFR* (intron 19)	GG	166	2.18 ± 1.03	90	1.74 ± 0.84b
		GT	262	2.21 ± 1.08	233	1.96 ± 0.81ab
		TT	109	2.23 ± 1.01	97	2.05 ± 0.82a
		*p*-value	0.915			0.0309*
*NGF*: g.91795933T>C	*NGF* (intron 2)	CC	115	2.32 ± 1.06	73	1.73 ± 0.79b
		CT	278	2.13 ± 1.05	194	1.94 ± 0.85ab
		TT	144	2.28 ±1.02	153	2.02 ± 0.79a
		*p*-value	0.157			0.0418*

Note: N and S.D. in table headers denote the sample size and standard deviation of litter size. a, b within the same column with different superscripts means *p* < 0.05. *p indicates the significant association at the significance level α = 0.05; **p indicates the significant association at the significance level α = 0.01.

**Table 4:** Litter size related quantitative trait loci (QTLs) annotation of nine significant single nucleotide polymorphisms (SNPs) associated with litter size in Hu sheep or Small-tailed Han sheep.

SNP ID	QTL ID	QTL Region (Chr:Mb)	Distance (Mb)	Reference
*KIT:* g.70199073A>G	13975	Chr6:68.3-68.4	1.80	[35]
	130449	Chr6: 42.6-42.6	27.60	[36]
	154661	Chr6: 29.4-29.4	40.80	[21]
	154662	Chr6:29.4-29.4	40.80	[21]
*KITLG*: g.124520653G>C	14242	Chr3:75.2-75.3	49.22	[37]
*ADAMTS1:* g.127753565T>C	-	-	-	-
*ADAMTS1*: g.127754640G>T	-	-	-	-
*NCOA1*: g.31928165C>T	14242	Chr3:75.2-75.3	43.27	[37]
*NCOA1*: g.32140565G>A	14242	Chr3:75.2-75.3	43.27	[37]
*LIFR:* g.35862868C>T	154682	Chr16:31.8-31.8	4.06	[21]
	154683	Chr16:31.8-31.8	4.06	[21]
	154684	Chr16:31.9-31.9	3.96	[21]
*LIFR:* g.35862947G>T	154682	Chr16:31.8-31.8	4.06	[21]
	154683	Chr16:31.8-31.8	4.06	[21]
	154684	Chr16:31.9-31.9	3.96	[21]
*NGF*: g.91795933T>C	-	-	-	-

Note: “-” denotes information is not available.

**Table 5:** Association between haplotype combinations and litter size in Small-tailed Han sheep.

Haplotype Combination (n)	Litter Size (Mean ± S.D.)	*p* Value
H1H1 (87)	1.70 ± 0.82b	0.0284*
H1H2 (214)	1.97 ± 0.81a	
H1H3 (17)	1.76 ± 0.75ab	
H2H2 (83)	2.02 ± 0.78a	
H2H3 (14)	2.21 ± 1.05a	

Note: S.D. in table headers denotes the standard deviation of litter size; H1denotes CG; H2 denotes TT; H3 denotes CT; a, b within the same column with different superscripts means *p* < 0.05; ^*^p indicates the significant association at the significance level α = 0.05.

## Supplementary Materials

The following are available online at https://www.mdpi.com/2076-2615/9/11/958/s1. Table S1: Primers and PCR condition applied for pooled-DNA sequencing for the ten candidate genes. Table S2: Information of 78 identified single nucleotide polymorphisms (SNPs) in the ten genes. Table S3: Associated between 36 single nucleotide polymorphisms (SNPs) with litter size in Hu sheep and Small-tailed Han sheep.
