# Supplementary material for: Association of Polymorphisms in Candidate Genes with the Litter Size in Two Sheep Breeds
Source: Animals (Basel). 2019 Nov 12;9(11):958. doi: 10.3390/ani9110958 (PMC6912326; doi:10.3390/ani9110958)
Supplement: Supplementary file 1 [file animals-09-00958-s001.zip › add_upload_Animals/Table S1.docx]

Association of polymorphisms in candidate genes with the litter size in two sheep breeds

Zehu Yuan, Junxia Zhang, Wanhong Li, Weiming Wang, Fadi Li and Xiangpeng Yue

**Table S1** Primers and PCR condition applied for pooled-DNA sequencing for the ten candidate genes

| Gene symbol  (NCBI gene ID) | Gene Name | Primers | Primer sequences（5′ -> 3′） | Gene region | Size of PCR products (bp) | Annealing temperature (°C) |
| --- | --- | --- | --- | --- | --- | --- |
| *NGF*  (101104540) | nerve growth factor | NGF1F  NGF1R | ACAGAGGGAAACACGAAT  TAGATGAGGTGCCCAGAC | 5'UTR, exon 1, intron1 | 1280 | 60 |
|  |  | NGF2F  NGF2R | GGCTCAGTGTCTAATCAT  TGTCCAGGGAGATACCAA | Intron1, exon2, intron2 | 599 | 60 |
|  |  | NGF3F  NGR3R | TCCCGGACTTCCGACCAT GGACACCACTGAGCCACT | Intron2, exon 3, 3'UTR | 1113 | 61 |
| *NTRK1*  (101109763) | neurotrophic receptor tyrosine kinase 1 | NTRK11F  NTRK11R | CGACTTAGAAGAGGAGAG  GTGGCTTGTGTTATCAGT | Intron1, exon 2, intron 2 | 533 | 56 |
|  |  | NTRK12F  NTRK12R | CTCTGTGCGTATGTGGAT  GAAATGGAAGGCATCTGG | Intron2, exon 3, intron3, exon 4 | 535 | 60 |
|  |  | NTRK13F  NTRK13R | CCAAGGAGGTATTTGAGGC  ATGGAAGCGAGACAGCAAG | Intron3, exon 4, intron 4 | 407 | 55 |
|  |  | NTRK14F  NTRK14R | CTCCCCTCGCTCACACTG  CCCAGCCTTTGCTTTGAT | Intron4, exon 5, intron 5 | 367 | 62 |
|  |  | NTRK15F  NTRK15R | CCCTCACCTGGCTTGTCA  CTCAACCTTCAGTTTCCC | Intron 5, exon 6, intron 6 | 621 | 61.6 |
|  |  | NTRK16F  NTRK16R | CAGGCAGGATTCGGAGGC  CCAAGGGTCTAAACCATCTCG | Intron6, exon 7, intron7 | 335 | 60 |
|  |  | NTRK17F  NTRK17R | ATGGGTGGCTTTATGGTTTG  ATCCCGCCCCTTTCCTGTAG | Intron7, exon 8, intron 8 | 507 | 63 |
|  |  | NTRK18F  NTRK18R | CTCCCGACTCTCACCGCC  CGAGGAAGCCAGCACAGA | Intron 8, exon 9, intron 9 | 543 | 61 |
|  |  | NTRK19F  NTRK19R | GGTTCTTTCTGGTCCCTG  CGAGTCCTGGCTGTGAAG | Exon 10, intron10, exon11, intron11 | 344 | 60 |
|  |  | NTRK110F  NTRK110R | CCTTCACAGCCAGGACTC  AAGAACCAGGAGGAAATAA | Intron11, exon12, intron12 | 320 | 61 |
|  |  | NTRK111F  NTRK111R | GGTTCTTCCCTTCTTCCTG  ACTGTCCCGCTGGGTTGT | Intron 12, exon 13, intron 13 | 504 | 51 |
|  |  | NTRK112F  NTRK112R | CCTGAACAAGCCTTTCCTCC  CATCCACCCCCAAGTCGT | Intron13, exon 14, intron 14 | 477 | 62 |
|  |  | NTRK113F  NTRK113R | CACGAAGTATTGGTGGTCC  ACGCACTCACTCCGCAAA | Intron 14, exon 15, intron 15 | 637 | 63 |
|  |  | NTRK114F  NTRK114R | AGATGCTGAGGCTGGAGT  CGGTAGTAGTCGGTGCTG | Intron15, Exon 16, intron 16 | 507 | 63 |
|  |  | NTRK115F  NTRK115R | TCTTTCACGAAGGTCCCA  CGGTAGTAGTCGGTGCTG | Exon 16 | 256 | 63 |
|  |  | NTRK116F  NTRK116R | GAGAGCCGCTTCTATTTAT  ACTGACCTCGGTGTTGGA | Intron 16, exon 17, intron 17 | 274 | 56 |
|  |  | NTRK117F  NTRK117R | TAATCCCTGTCACTGCTC  CCTGTCCACCTAACTTCA | Intron17, exon18, 3'UTR | 909 | 51 |
| *KITLG*  (443371) | KIT ligand | KITLG1F  KITLG1R | AAGTCCCCACCCATCTCT  CCTCCCTTTTACAGACAGC | 5'UTR, exon 1, intron 1 | 202 | 52 |
|  |  | KITLG2F  KITLG2R | GGAATAACACTGCTTGCC  GTTGAAGTCCTCCCTGTAG | Intron 1, Exon 2, Intron 2 | 407 | 61 |
|  |  | KITLG3F  KITLG3R | GTGGGACTGAAGAGACAA  CTCCAACATCCTAAAACTT | Intron 2, exon 3, intron 3 | 412 | 54 |
|  |  | KITLG4F  KITLG4R | CCAAACTAAGGAGGGACG  GGAACAACCGCAGCATTA | Intron 3, exon 4, intron 4 | 389 | 52 |
|  |  | KITLG5F  KITLG5R | TTATCTCCAGCTTTAGGG  AAGGTGACTTTGGTGAAT | Intron 4, exon 5, intron 5 | 468 | 60 |
|  |  | KITLG6F  KITLG6R | TTCAGTCTTGCTTCTTACA  GTTGTCTAAAAGTGTCCG | Intron 5, exon 6, intron 6 | 329 | 57 |
|  |  | KITLG7F  KITLG7R | ACATTTATTTTCTGTGCTC  AACAGGTGCTTTGTCTTT | Intron 6, exon 7, intron 7 | 352 | 55 |
|  |  | KITLG8F  KITLG8R | GCTACCACAATGAAACCC  CTGGAGAGTGCTTTTGAT | Intron 7, exon 8, intron 8 | 296 | 54 |
|  |  | KITLG9F  KITLG9R | AACAAGATGCTCCGTGAC  CTTCAACTGCTCCTATTT | Intron 8, exon 9, intron 9 | 261 | 56 |
|  |  | KITLG10F  KITLG10R | CGGGCACGGTTTTTCTTG  TCCCTAAAGCAGGACGAG | Intron 9, exon 10, 3'UTR | 701 | 60 |
| *KIT*  (780504) | KIT proto-oncogene, receptor tyrosine kinase | KIT1F  KIT1R | AGTTACAGTGCAACCAGT  CCTTCAAACCTCCCACAC | 5'UTR, exon 1, intron 2 | 246 | 52.7 |
|  |  | KIT2F  KIT2R | GAATATAAATTGTATGGTA  AGTGATACTAAAATCCCTA | Intron 1, exon 2, intron 2 | 346 | 44.6 |
|  |  | KIT3F  KIT3R | CTGGAAATCAGTCAGTTACCTT  ATGAAACAACAAGTGAAG | Intron 2, exon 3, intron 3 | 354 | 56.3 |
|  |  | KIT4F  KIT4R | GGAAACTGTGAAAAAGCCA  CAAACACCAGCCACAACTTA | Intron 3, exon 4, intron 4 | 255 | 48.2 |
|  |  | KIT5F  KIT5R | CACAGGTGACTGATGGGT  TCAATGGTTGGCTCCGGGAA | Intron 4, exon 5, intron 5 | 535 | 57.3 |
|  |  | KIT6F  KIT6R | AAAGTATGCCGTATCCAATGC  CAGAGTCTAAACATCCCCT | Intron 5, exon 6, intron 6 | 312 | 51.6 |
|  |  | KIT7F  KIT7R | CTGTGATGGGTGGTAGGTA  AGCATTTGAGGGAGAGAAC | Intron 6, exon 7, intron 7 | 252 | 59.8 |
|  |  | KIT8F  KIT8R | GGTGAGTTGGAGGTGAGA  CTATGTGAAAACAAAAAGG | Intron 7, exon 8, intron 8 | 664 | 53.7 |
|  |  | KIT9F  KIT9R | TTGGTAGATTTAGTATCAGGTA  AGTTCAGACATTAGGGCTT | Intron 8, exon 9, intron 9 | 373 | 53.7 |
|  |  | KIT10F  KIT10R | TGGGAAAGTCGTTGAGGC  TTGACCAATTACAAGAGGCAGG | Intron 9, exon 10, intron 10 | 313 | 55.8 |
|  |  | KIT11F  KIT11R | ACTCGTGCTTGGTAGGCTTA  CTTGATTGCAAACCCTTATGAC | Intron 10, exon 11, intron 11 | 346 | 50.3 |
|  |  | KIT12F  KIT12R | GGGAGTGGAGAGATAGCC  CTTACCCTGAGCCTATTG | Intron 11, exon 12, intron 12 | 302 | 56.1 |
|  |  | KIT13F  KIT13R | GTAACTTAGCCCAGGATTG  CACTTCAAGCGACACTCTG | Intron 12, exon 13, intron 13 | 418 | 57.5 |
|  |  | KIT14F  KIT14R | TAAGCGGCATTCTAGCATT  CTGTCAAGAGAGAGTGGGTA | Intron 13, exon 14, intron 14 | 241 | 57.3 |
|  |  | KIT15F  KIT15R | GCCCATCAAGTTCTCACC  GGCAAGGCTCACTTTACC | Intron 14, exon 15, intron 15 | 249 | 52.6 |
|  |  | KIT16F  KIT16R | TACCAGTGCTCTCCTTGCTT  TGAAAACCCTCAACATACGG | Intron 15, exon 16, intron 16 | 245 | 58.2 |
|  |  | KIT17F  KIT17R | TAAAGTTTTGGCGGAGTC  AGAGAAAGGGTGGGTAGC | Intron 16, exon 17, intron 17 | 375 | 57.8 |
|  |  | KIT18-1F  KIT18-1R | GTGTGGTTCAGTTCTTGG  TCAGGAGCAGAGAGCATT | Exon 18, intron 18 | 801 | 57.3 |
|  |  | KIT18-2F  KIT18-2R | AATCTAGTATTTCATGCTG  ATAAGGTCTAAGGAACAGGT | Exon 18 | 857 | 57.8 |
| *LIF*  (101110180) | LIF interleukin 6 family cytokine | LIF1-1F  LIF1-1R | CAAAAGAAACAGCGGCAG  TACTTGGGTGAGACTGGG | 5'UTR, exon 1 | 928 | 54.9 |
|  |  | LIF1-2F  LIF1-2R | ACTCCCCAGTCTCACCCA  TCCCTGCCTCCCACTTCA | Exon 1, intron 1 | 993 | 54.7 |
|  |  | LIF2F  LIF2R | CCTTCCCTTCTCTGACCA  CTCTCCACCTCTTGTCCG | Intron 1, exon 2, intron 2 | 298 | 52.2 |
|  |  | LIF3F  LIF3R | CGGCAGTTTTCAGAGGTT  CCAGGTGAGTCAGGGTTG | Intron 2, exon 3, intron 3 | 396 | 53 |
|  |  | LIF4F  LIF4R | GAAAAGGGAACTTGAGGG  TCCAGAGAAGGTTCAGCG | Exon 4, 3'UTR | 480 | 51.5 |
| *LIFR*  (101106364) | LIF receptor alpha | LIFR1-1F  LIFR1-1R | CTAAGCACAGCACGTCAT  TACCTGTCAGCCTACCCA | 5'UTR, exon 1 | 1127 | 48.9 |
|  |  | LIFR1-2F  LIFR1-2R | CAGGTGGCTCAGTGGTAG  AAGATCAGGGAAAGAGTG | Intron 1, exon 1 | 861 | 50.7 |
|  |  | LIFR2F  LIFR2R | CTGGGGATGGTTTTGACT  TGTTTCCACCCTGACTGA | Intron 1, exon 2, intron 2 | 366 | 52.2 |
|  |  | LIFR3F  LIFR3R | ACTGACCACTTTCCTCTC  GAGTTTGCTTTCATCGTG | Intron 2, exon 3, intron 3 | 495 | 45 |
|  |  | LIFR4F  LIFR4R | TCAAGAAGGAATAGAGTT  GTCATAGCATACAGTTTT | Intron 3, exon 4, intron 4 | 404 | 40.2 |
|  |  | LIFR5F  LIFR5R | GATACAGTTCATCATTGG  AAATAGGCACTTACAAGA | Intron 4, exon 5, intron 5 | 159 | 41.4 |
|  |  | LIFR6F  LIFR6R | CCCCACTATCACCCTATC  ACCTGAACTTGCTTGAAAA | Intron 5, exon 6, intron 6 | 544 | 49 |
|  |  | LIFR7F  LIFR7R | AGATCCACCAGACATTCC  TTCCAGTGTGCTCCTTAC | Intron 6, exon 7, intron 7 | 180 | 48.5 |
|  |  | LIFR8F  LIFR8R | AGCCGAGAAAGAAGAACC  GGAACACACGAATGCCAA | intron 7, exon 8, intron 8 | 555 | 51.4 |
|  |  | LIFR9F  LIFR9R | CAAACACTGGGTGGAACA  CACACAATGGCTCTTAGG | Intron 8, exon 9, intron 9 | 546 | 51.7 |
|  |  | LIFR10F  LIFR10R | TTTGGGAGGGTTTCTTCT  CCTACTTTTAGTCACTCTGG | Intron 9, exon 10, 3'UTR | 362 | 51.5 |
|  |  | LIFR11F  LIFR11R | TGTGAGATGGAGAGGCAGC  CCCTAATAACCCAAGAACC | Intron 10, exon 11, intron 11 | 419 | 56.1 |
|  |  | LIFR12F  LIFR12R | CTTGTCACATCACCTTTG  ACCTTGGTTTCCTGTCCT | Intron 11, exon 12, intron 12 | 319 | 45.4 |
|  |  | LIFR13F  LIFR13R | TTGGAATGGGAAATAGGA  TGGTTAGCCGCTGTAGTG | Intron 12, exon 13, intron 13 | 297 | 50.5 |
|  |  | LIFR14F  LIFR14R | ACCATTTTTTCCCCCTTC  ACTGATCTACAGGATGAAC | Intron 13, exon 14, intron 14 | 264 | 53.7 |
|  |  | LIFR15F  LIFR15R | GTTCTACCCTTGTATCTTCA  GTTTTGGAGACCAGTTTGA | Intron 14, exon 15, intron 15 | 484 | 46.5 |
|  |  | LIFR16F  LIFR16R | ATAACCCTGTATGCGAGAC  CTTTCAACACTGCCCTCC | Intron 15, exon 16, intron 16 | 493 | 49.5 |
|  |  | LIFR17F  LIFR17R | GGGAAACCCTGCTCTAAA  GTCGCAAGTTGTGCCAGA | Intron 16, exon 17, intron 17 | 371 | 52.3 |
|  |  | LIFR18F  LIFR18R | GTTCTAGCAACCGATCAA  AGCAGATGGTCAAGCACT | Intron 17, exon 18, intron 18 | 373 | 48.2 |
|  |  | LIFR19-1F  LIFR19-1R | TTTGTAGAAGGAGCATCG  TTGAAGGCAGAGTAGTGTG | Intron 18, exon 19 | 954 | 48.5 |
|  |  | LIFR19-2F  LIFR19-2R | CTGCTCTGTCGTTCATCT  CTTCAATCTGCTTCTCCA | Exon19 | 995 | 47.3 |
|  |  | LIFR19-3F  LIFR19-3R | TGAGTGTGGTTCTTGGAG  GAGCCGAACAAAGGAGTC | Exon 19, 3'UTR | 827 | 47.7 |
| *ADAMTS1*  (101107392) | ADAM metallopeptidase with thrombospondin type 1 motif 1 | ADAMTS1F  ADAMTS1R | CGGACCCGAACAATAGCC  CCCTCAAGTCGTTCACCC | 5'UTR, exon 1 | 872 | 58.5 |
|  |  | ADAMTS2F  ADAMTS2R | TTGACTGGTTAGGCTTTC  TGTCTGGTGAATAGAATCG | Intron 1, exon 2, intron | 470 | 44.6 |
|  |  | ADAMTS3F  ADAMTS3R | AGGGCTACTCAGCAACAA  AACTCGCACTCTGGAATA | Intron 2, exon 3, intron 3 | 285 | 50.7 |
|  |  | ADAMTS4F  ADAMTS4R | CACCCTGTGAAAAGTAGAC  GAAATGTTGTAATCGTGGA | Intron 3, exon 4, intron 4 | 305 | 46.7 |
|  |  | ADAMTS5F  ADAMTS5R | TCTCCCAAACCCTTCAGC  CCTCTATGACAACCCTTT | Intron 4, exon 5, intron 5 | 610 | 55.2 |
|  |  | ADAMTS6F  ADAMTS6R | CTCAGACATTTGATGCCAC  ACACTGTTCCAGCCTAAA | Intron 5, exon 6, intron 6 | 461 | 50.9 |
|  |  | ADAMTS7F  ADAMTS7R | CGAAGAACGGAGGAAGATA  TCAAAGCAGGCAGTTACCA | Intron 6, exon 7, intron 7 | 594 | 52.6 |
|  |  | ADAMTS8F  ADAMTS8R | GCTCTGCTTTCGTTGACC  CTCTGAGTCCGTTGCTTC | intron 7, exon 8, intron 8 | 662 | 53.3 |
|  |  | ADAMTS9-1F  ADAMTS9-1R | TTCTTACCTGATTGCCTGG  TTTCTTGTGCTACCTCATTGG | Intron 8, exon 9 | 1023 | 53.3 |
|  |  | ADAMTS9-2F  ADAMTS9-2R | ATGGAGGAAGGTGAGATT  AATAGCACGGCTGAGTAG | Exon19, 3'UTR | 1232 | 47.8 |
| *NCOA1*  (101107264) | nuclear receptor coactivator 1 | NCOA1F  NCOA1R | GAGCGTTCCCGTAAGCCC  CGGTTTCTCCCATTACTCTT | 5'UTR, exon 1, intron 1 | 300 | 60.3 |
|  |  | NCOA2F  NCOA2R | ATAAAGCATTTGTGTGAGA  ATACACTGACTGCCAAGA | Intron 1, exon 2, intron 2 | 276 | 45.4 |
|  |  | NCOA3F  NCOA3R | ATCTAACCTCCCTTCTGCT  CTCCGTAGGGTAGTTTCC | Intron 2, exon 3, intron 3 | 152 | 46.9 |
|  |  | NCOA4F  NCOA4R | TTACAGGGATGGAGAAGA  CACGAATAGAGGAGGTTT | Intron 3, exon 4, intron 4 | 586 | 47.1 |
|  |  | NCOA5F  NCOA5R | TGTTTTCTGCTCTTTGGC  CTGACTCTTCTGGGTTTC | Intron 4, exon 5, intron 5 | 593 | 51.4 |
|  |  | NCOA6F  NCOA6R | GCTCCCACTTATTAGTTGTA  GAAACAGCAAAGCCAGAC | Intron 5, exon 6, intron 6 | 585 | 47.3 |
|  |  | NCOA7F  NCOA7R | AAATCCTGTTCCCTCCAC  TCTGCTTCCTAACTCTGTCA | Intron 6, exon 7, intron 7 | 597 | 51.2 |
|  |  | NCOA8F  NCOA8R | TTTGCCTCAGGATAGTGT  TTTAGTGGGAATGTCAGC | intron 7, exon 8, intron 8 | 571 | 47.4 |
|  |  | NCOA9F  NCOA9R | TATTCAGTGGGAAGGGAT  AGGGGACAGTCACATTCA | Intron 8, exon 9, intron 9 | 384 | 49.1 |
|  |  | NCOA10F  NCOA10R | CTTTGGGTCTTTGTGGTA  CTTCCTTTCCTAAAACTGA | Intron 9, exon 10, 3'UTR | 560 | 48.1 |
|  |  | NCOA11F  NCOA11R | GAGGCACCCATTCAAGTA  CCACACCTTTCCTTTCTCC | Intron 10, exon 11, intron 11 | 601 | 50.6 |
|  |  | NCOA12F  NCOA12R | ATGGAAACCAATGACGATA  AGAGGCAGAGGTGGAAGT | Intron 11, exon 12, intron 12 | 951 | 50.5 |
|  |  | NCOA13F  NCOA13R | AGATGAAAGTGTAGAATGT  CTACTCTGGAAGGGTTGG | Intron 12, exon 13, intron 13 | 511 | 49.3 |
|  |  | NCOA14F  NCOA14R | CCTTTGTATCATTGAGGTAG  TTCTGGCTTATTCTGGTTTA | Intron 13, exon 14, intron 14 | 250 | 48.8 |
|  |  | NCOA15F  NCOA15R | CGAAGGAAGAAATGATGA  GCTACCACACAGTGAGAG | Intron 14, exon 15, intron 15 | 226 | 48.0 |
|  |  | NCOA16F  NCOA16R | GTTGATTCTTAGGCTTTG  TACTCACCTGCTGTTGTC | Intron 15, exon 16, intron 16 | 605 | 44.8 |
|  |  | NCOA17F  NCOA17R | GACCAAAGCGATTTAGCA  TTCAGGACAGCAATAAGAG | Intron 16, exon 17, intron 17 | 586 | 51.7 |
|  |  | NCOA18F  NCOA18R | TCCAAGGTAAGGGGTAATC  ATCCACGATGCCCAAGTA | Intron 17, exon 18, intron 18 | 766 | 51.5 |
|  |  | NCOA19F  NCOA19R | TGATAGACCCTTCTGGAT  GAGTATTGGGATGCTGAT | Intron 18, exon 19, intron 19 | 297 | 45.9 |
|  |  | NCOA20F  NCOA20R | ACCCTTTGGATAGAGTTC  GCTGTGTCACTTACCTGCT | Intron 19, exon 20, intron 20 | 400 | 45.2 |
|  |  | NCOA21F  NCOA21R | CCAGAGGTTCTCCCGTTAC  GCAGAGCCAGGACTCACAC | Intron 20, exon 21, intron 21 | 430 | 54.2 |
|  |  | NCOA22F  NCOA22R | GGAACTCAAAAACCCACGG  CCTTCACCCATCTCATCAA | Intron 21, exon 22, 3'UTR | 797 | 57.6 |
| *NPM1*  (101104783) | nucleophosmin 1 | NPM1F  NPM1R | CATTGTTAGGATGGGTTTG  TTGCCAAGTGGTAAAGAGG | 5'UTR, exon 1, intron1 | 541 | 50.8 |
|  |  | NPM2F  NPM2R | TCCCCACAGACAGCCTAT  ACAAGCAAACCCATCAGC | Intron 1, exon 2, intron 2 | 591 | 52.6 |
|  |  | NPM3F  NPM3R | ATTTGGCTGATGGGTTTG  CTCCTCTTCTTCTGACTCTG | Intron 2, exon 3, intron 3 | 445 | 63.5 |
|  |  | NPM4F  NPM4R | CTGTGGTCTTACGGTTGA  CTCCAGGGCTTACTACTT | Intron 3, exon 4, intron 4 | 555 | 48.4 |
|  |  | NPM5F  NPM5R | TCTGGTGCTCCTGTCTGT  GTATCCACTAAATCCTCCCC | Intron 4, exon 5, intron 5 | 580 | 50.1 |
|  |  | NPM6F  NPM6R | GATAAGGAGAATCGCACAG  TAAAAGCAGCAGTCAGGT | Intron 5, exon 6, intron 6 | 568 | 50.0 |
|  |  | NPM7F  NPM7R | GATGCCATTGCTTGTTTG  CTTACTGTCAGCCCTCCAC | Intron 6, exon 7, intron 7 | 665 | 52.3 |
|  |  | NPM8F  NPM8R | TTATCACATAGGGCAGTT  ATAGAACAGGCAATGGGA | intron 7, exon 8, intron 8 | 663 | 45.0 |
|  |  | NPM9F  NPM9R | TTTAGAGTGGTTGGGAGG  TGGAGCAGCAAGGAAAAC | Intron 8, exon 9, intron 9 | 630 | 49.9 |
|  |  | NPM10F  NPM10R | CAAAGTGGAAGCCAAGTT  TGGGTGGTAGGAAGGTCT | Intron 9, exon 10, 3'UTR | 506 | 503 |
| *NOG*  (780490) | noggin | NOGGIN1F  NOGGIN1R | GGATCTGAACGAGACGCTG  ATGATGGGGACCTGGATG | 5'UTR, exon 1, 3'UTR | 498 | 55.9 |
