# Supplementary material for: Association of Polymorphisms in Candidate Genes with the Litter Size in Two Sheep Breeds
Source: Animals (Basel). 2019 Nov 12;9(11):958. doi: 10.3390/ani9110958 (PMC6912326; doi:10.3390/ani9110958)
Supplement: Supplementary file 1 [file animals-09-00958-s001.zip › add_upload_Animals/Table S2.docx]

Association of polymorphisms in candidate genes with the litter size in two sheep breeds

Zehu Yuan, Junxia Zhang, Wanhong Li, Weiming Wang, Fadi Li and Xiangpeng Yue

**Table S2** Information of 78 identified single nucleotide polymorphisms (SNPs) in the ten candidate genes.

| **SNP ID** | **SNPscan** | **Gene Symbol** | **Chr.** | **Exon/**  **Intron** | **Amino acid substitution** | **Sequencing results of pooled DNA** |
| --- | --- | --- | --- | --- | --- | --- |
| g.70199073A>G | Yes | *KIT* | 6 | intron 2 |  | 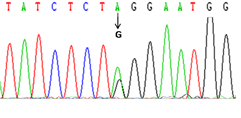 |
| g.70224398T>A | Yes |  |  | exon 10 | Leu→His | 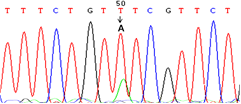 |
| g.70252062G>A | No |  |  | intron 18 |  | 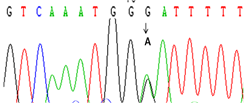 |
| g.124501271T>C | No | *KITLG* | 3 | intron 2 |  | 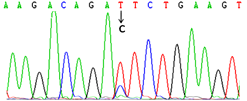 |
| g.124502403C>T | No |  |  | intron 2 |  | 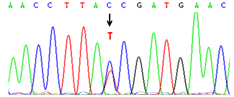 |
| g.124502568G>T | No |  |  | intron 3 |  | 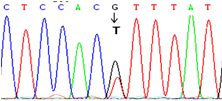 |
| g.124509908T>C | No |  |  | intron 5 |  | 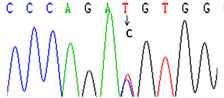 |
| g.124511398T>C | No |  |  | intron 5 |  | 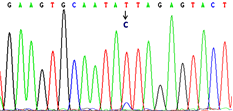 |
| g.124520653G>C | Yes |  |  | intron 9 |  | 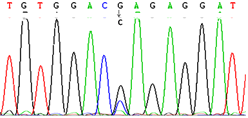 |
| g.127751615C>T | Yes | *ADAMTS1* | 1 | exon 2 | Ala→Ala | 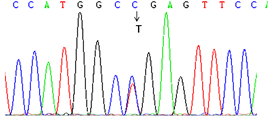 |
| g.127753565T>C | Yes |  |  | exon 5 | Ser→Ser | 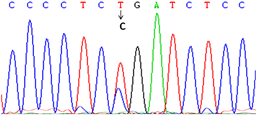 |
| g.127753643C>T | Yes |  |  | exon 5 | Asp→Asp | 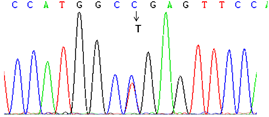 |
| g.127753727C>T | Yes |  |  | exon 5 | Ala→Ala | 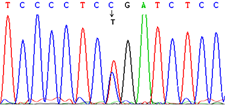 |
| g.127754640T>G | Yes |  |  | intron 7 |  | 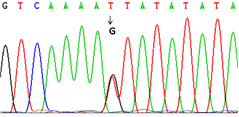 |
| g.127755231C>T | No |  |  | intron 8 |  | 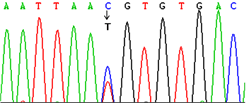 |
| g.127756130G>A | Yes |  |  | exon 9 | Met→Val | 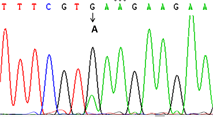 |
| g.31928165C>T | Yes | *NCOA1* | 3 | intron 1 |  | 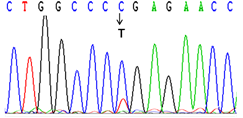 |
| g.31928230C>T | Yes |  |  | intron 1 |  | 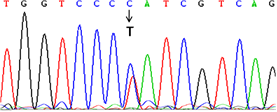 |
| g.32072394C>T | Yes |  |  | exon 8 | Pro→Pro | 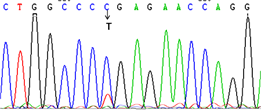 |
| g.32116034A>G | Yes |  |  | intron 17 |  | 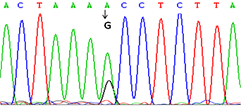 |
| g.32140565G>A | Yes |  |  | intron 21 |  | 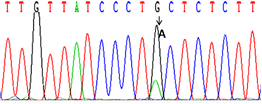 |
| g.32140837T>C | Yes |  |  | intron 21 |  | 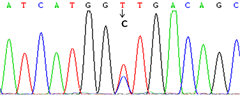 |
| g.3245714T>C | Yes | *NPM1* | 16 | intron 1 |  | 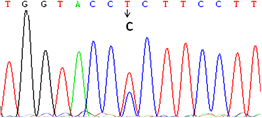 |
| g.3245741C>T | Yes |  |  | intron 1 |  | 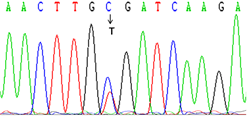 |
| g.3245965C>T | Yes |  |  | intron 2 |  | 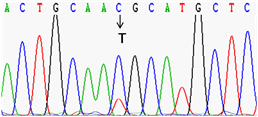 |
| g.3245996T>C | No |  |  | intron 2 |  | 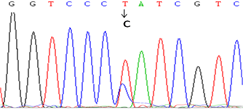 |
| g.3246266T>G | Yes |  |  | intron 3 |  | 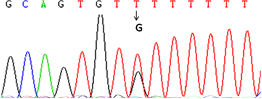 |
| g.3246852G>A | No |  |  | intron 4 |  | 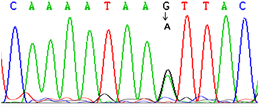 |
| g.3247135T>G | Yes |  |  | intron 5 |  | 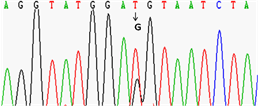 |
| g.3247326T>C | No |  |  | intron 5 |  | 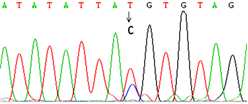 |
| g.3247450C>T | Yes |  |  | intron 5 |  | 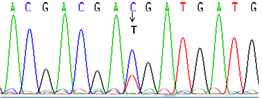 |
| g.3247499A>T | Yes |  |  | intron 5 |  | 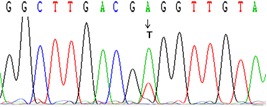 |
| g.3247689T>A | No |  |  | exon 5 | Asp→Asp | 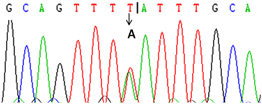 |
| g.3247907T>C | No |  |  | intron 6 |  | 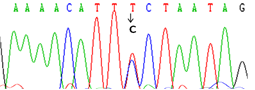 |
| g.3250910T>C | No |  |  | intron 7 |  | 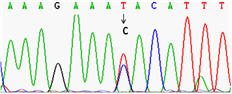 |
| g.3251189A>T | Yes |  |  | intron 7 |  | 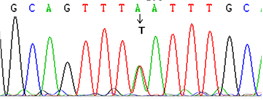 |
| g.68801067C>T | Yes | *LIF* | 17 | intron 1 |  | 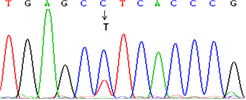 |
| g.68816215C>T | Yes |  |  | intron 4 |  | 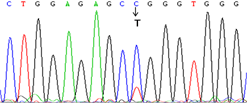 |
| g.35813711C>T | Yes | *LIFR* | 16 | intron 1 |  | 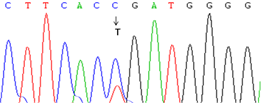 |
| g.35814094C>T | Yes |  |  | intron 1 |  | 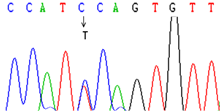 |
| g.35813931G>A | Yes |  |  | intron 1 |  | 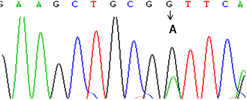 |
| g.35813935A>G | Yes |  |  | intron 1 |  | 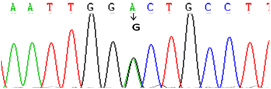 |
| g.35817147A>G | Yes |  |  | intron 2 |  | 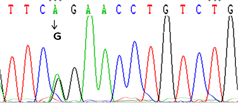 |
| g.35817247G>A | Yes |  |  | intron 2 |  | 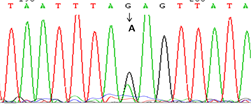 |
| g.35835329G>A | Yes |  |  | exon 6 | Ala→Ala | 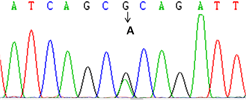 |
| g.35835474G>T | Yes |  |  | exon 7 | Ala→Ser | 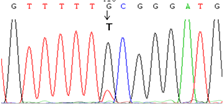 |
| g.35841608T>C | Yes |  |  | intron 9 |  | 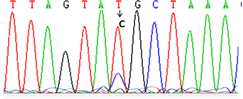 |
| g.35845474G>A | NO |  |  | intron 11 |  | 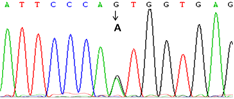 |
| g.35845633T>C | Yes |  |  | intron 11 |  | 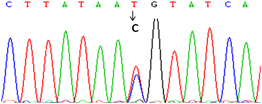 |
| g.35847837A>G | Yes |  |  | intron 12 |  | 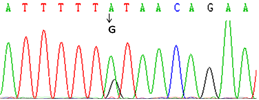 |
| g.35847864A>T | Yes |  |  | exon 12 | Asn→Ser | 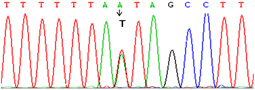 |
| g.35848079G>A | Yes |  |  | intron 12 |  | 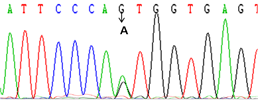 |
| g.35848108A>G | Yes |  |  | intron 13 |  | 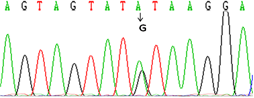 |
| g.35847912C>T | Yes |  |  | intron 13 |  | 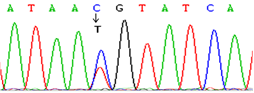 |
| g.35851829T>C | Yes |  |  | intron 14 |  | 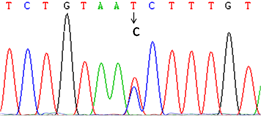 |
| g.35853589T>C | Yes |  |  | intron 14 |  | 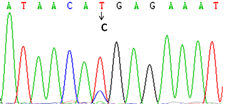 |
| g.35853637T>G | Yes |  |  | intron 14 |  | 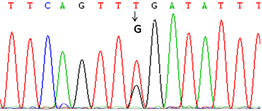 |
| g.35853852T>C | Yes |  |  | intron 19 |  | 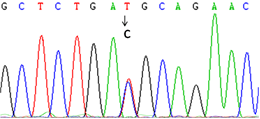 |
| g.35862868C>T | Yes |  |  | intron 19 |  | 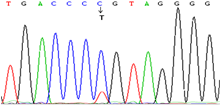 |
| g.35862947G>T | Yes |  |  | intron 19 |  | 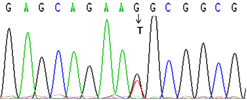 |
| g.35867028T>C | Yes |  |  | intron 19 |  | 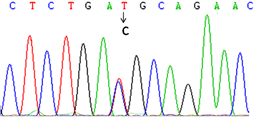 |
| g.91651197G>A | No | *NGF* | 1 | exon 1 | Ala→Ala | 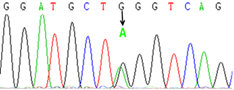 |
| g.91787324C>A | No |  |  | intron 1 |  | 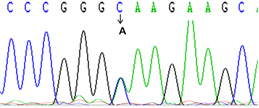 |
| g.91789543A>T | No |  |  | intron 1 |  | 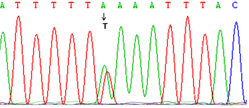 |
| g.91795933T>C | Yes |  |  | intron 2 |  | 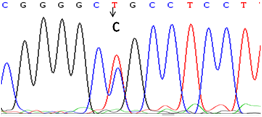 |
| g.105274029C>T | No | *NTRK1* | 1 | intron 2 |  | 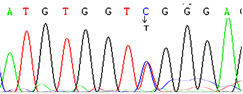 |
| g.105274304G>A | No |  |  | intron 2 |  | 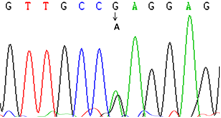 |
| g.105274589T>C | No |  |  | intron 3 |  | 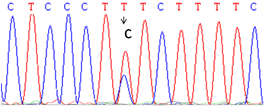 |
| g.105276945C>T | Yes |  |  | intron 5 |  | 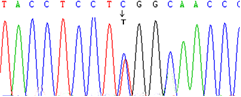 |
| g.105278973G>C | No |  |  | intron 7 |  | 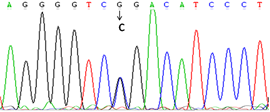 |
| g.105279563G>A | No |  |  | intron 7 |  | 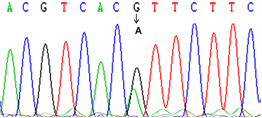 |
| g.105280119T>C | No |  |  | intron 7 |  | 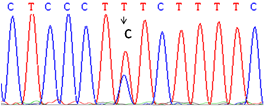 |
| g.105281586C>T | No |  |  | intron 9 |  | 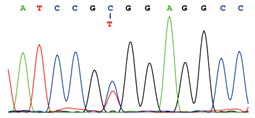 |
| g.105281965T>C | No |  |  | intron 9 |  | 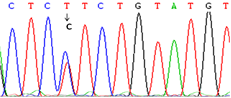 |
| g.105282108G>A | No |  |  | intron 10 |  | 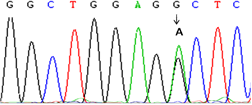 |
| g.105283128T>C | No |  |  | intron 12 |  | 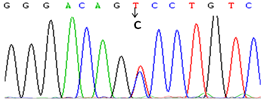 |
| g.105284246G>C | No |  |  | intron 14 |  | 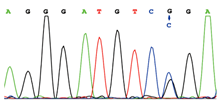 |
| g.105288550C>G | Yes |  |  | intron 14 |  | 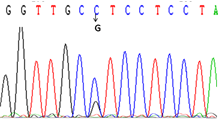 |
