# Supplementary material for: Association of Polymorphisms in Candidate Genes with the Litter Size in Two Sheep Breeds
Source: Animals (Basel). 2019 Nov 12;9(11):958. doi: 10.3390/ani9110958 (PMC6912326; doi:10.3390/ani9110958)
Supplement: Supplementary file 1 [file animals-09-00958-s001.zip › add_upload_Animals/Table S3 .docx]

Association of polymorphisms in candidate genes with the litter size in two sheep breeds

Zehu Yuan, Junxiang Zhang, Wanhong Li, Weiming Wang, Fadi Li, Xiangpeng Yue

**Table S3.** Associated between 36 single nucleotide polymorphisms (SNPs) with litter size in Hu sheep and Small-tailed Han sheep.

| SNP ID | Genotype | Hu Sheep | | | Small-tailed Han sheep | | |
| --- | --- | --- | --- | --- | --- | --- | --- |
|  |  | n | Litter Size (Mean ± S.D.) | P value | n | Litter Size (Mean ± S.D.) | P value |
| g.127751615C>T | CC | - 1 | - | - | 333 | 1.91 ± 0.83 | 0.611 |
|  | CT | - | - |  | 81 | 2.01 ± 0.78 |  |
|  | TT | - | - |  | 6 | 2.00 ± 1.10 |  |
| g.127753643C>T | CC | - | - | - | 271 | 1.93 ± 0.84 | 0.318 |
|  | CT | - | - |  | 130 | 1.91 ± 0.81 |  |
|  | TT | - | - |  | 19 | 2.21 ± 0.54 |  |
| g.31928230C>T | CC | 379 | 2.18 ± 1.06 | 0.165 | 237 | 1.92 ± 0.86 | 0.92 |
|  | CT | 145 | 2.30 ± 1.01 |  | 148 | 1.96 ± 0.79 |  |
|  | TT | 10 | 1.7 ± 0.82 |  | 32 | 1.94 ± 0.76 |  |
| g.32072394C>T | CC | 355 | 2.21 ± 1.08 | 0.374 | 251 | 1.9 ± 0.85 | 0.196 |
|  | CT | 159 | 2.23 ± 0.98 |  | 153 | 2.01 ± 0.79 |  |
|  | TT | 23 | 1.91 ± 1.08 |  | 16 | 1.69 ± 0.60 |  |
| g.32116034A>G | AA | 331 | 2.18 ±1.06 | 0.388 | 265 | 1.89 ± 0.85 | 0.0647 |
|  | GA | 174 | 2.28 ±1.02 |  | 140 | 2.04 ± 0.78 |  |
|  | GG | 31 | 2.03 ± 1.02 |  | 14 | 1.57 ± 0.51 |  |
| g.32140837T>C | CC | 36 | 2.08 ± 0.99 | 0.411 | 19 | 1.68 ± 0.58 | 0.195 |
|  | CT | 181 | 2.29 ± 1.02 |  | 159 | 2.01 ± 0.79 |  |
|  | TT | 318 | 2.18 ± 1.06 |  | 241 | 1.90 ± 0.86 |  |
| g.3245714T>C | CC | 100 | 2.12 ± 1.01 | 0.204 | 134 | 1.92 ± 0.79 | 0.945 |
|  | CT | 260 | 2.29 ± 1.07 |  | 191 | 1.95 ± 0.85 |  |
|  | TT | 175 | 2.14 ± 1.04 |  | 94 | 1.93 ± 0.83 |  |
| g.3245741C>T | CC | 174 | 2.16 ± 1.04 | 0.344 | 94 | 1.93 ± 0.83 | 0.866 |
|  | CT | 266 | 2.27 ± 1.06 |  | 199 | 1.95 ± 0.84 |  |
|  | TT | 97 | 2.12 ±1.02 |  | 127 | 1.91 ± 0.79 |  |
| g.3245965C>T | CC | 171 | 2.16±1.04 | 0.27 | 93 | 1.91 ± 0.83 | 0.857 |
|  | CT | 265 | 2.28 ±1.06 |  | 190 | 1.96 ± 0.85 |  |
|  | TT | 101 | 2.09 ±1.02 |  | 137 | 1.91 ± 0.78 |  |
| g.3246266T>G | GG | 38 | 2.11 ± 1.06 | 0.654 | 26 | 2.04 ± 0.72 | 0.404 |
|  | GT | 180 | 2.17 ± 0.99 |  | 119 | 2.00 ± 0.82 |  |
|  | TT | 318 | 2.24 ± 1.08 |  | 274 | 1.89 ± 0.83 |  |
| g.3247499A>T | AA | 262 | 2.17± 1.01 | 0.703 | 204 | 1.93 ± 0.77 | 0.98 |
|  | AT | 233 | 2.34 ±1.10 |  | 171 | 1.98 ± 0.89 |  |
|  | TT | 38 | 2.89 ±1.01 |  | 43 | 1.95 ± 0.82 |  |
| g.3251189A>T | AA | 179 | 2.14 ± 1.04 | 0.244 | 95 | 1.94 ± 0.84 | 0.886 |
|  | AT | 262 | 2.29 ±1.06 |  | 200 | 1.95 ± 0.84 |  |
|  | TT | 96 | 2.13 ± 1.03 |  | 125 | 1.90 ± 0.79 |  |
| g.68801067C>T | CC | - | - | - | 326 | 1.94 ± 0.83 | 0.813 |
|  | CT | - | - |  | 84 | 1.92 ± 0.81 |  |
|  | TT | - | - |  | 9 | 1.78 ± 0.67 |  |
| g.68816215C>T | CC | 472 | 2.21 ± 1.06 | 0.749 | 334 | 1.96 ± 0.84 | 0.396 |
|  | CT | 64 | 2.22 ± 1.00 |  | 81 | 1.83 ± 0.74 |  |
|  | TT | 1 | 3.0 ± - |  | 5 | 1.80 ± 0.84 |  |
| g.35813711C>T | CC | 172 | 2.16 ± 0.98 | 0.211 | 194 | 1.95 ± 0.86 | 0.809 |
|  | CT | 269 | 2.19± 1.05 |  | 184 | 1.93 ± 0.79 |  |
|  | TT | 95 | 2.38 ± 1.14 |  | 42 | 1.86 ± 0.81 |  |
| g.35814094C>T | CC | 170 | 2.17 ± 0.98 | 0.309 | 190 | 1.94 ± 0.86 | 0.832 |
|  | CT | 272 | 2.18 ± 1.05 |  | 185 | 1.92 ± 0.79 |  |
|  | TT | 95 | 2.36 ±1.48 |  | 42 | 1.86 ± 0.81 |  |
| g.35813931C>T | AA | 26 | 2.08 ± 0.79 | 0.803 | 43 | 2.02 ± 0.83 | 0.282 |
|  | AG | 152 | 2.22 ± 1.02 |  | 155 | 1.99 ± 0.82 |  |
|  | GG | 356 | 2.21 ± 1.08 |  | 220 | 1.87 ± 0.82 |  |
| g.35813935A>G | AA | 166 | 2.17 ± 0.98 | 0.24 | 186 | 1.94 ± 0.85 | 0.942 |
|  | AG | 252 | 2.16 ± 1.04 |  | 170 | 1.94 ± 0.80 |  |
|  | GG | 116 | 2.35 ± 1.16 |  | 61 | 1.90 ± 0.81 |  |
| g.35817147A>G | AA | 372 | 2.20 + 1.06 | 0.778 | 222 | 1.89 ± 0.82 | 0.406 |
|  | AG | 146 | 2.25 ± 1.05 |  | 167 | 1.97 ± 0.82 |  |
|  | GG | 19 | 2.11 ± 0.81 |  | 31 | 2.06 ± 0.85 |  |
| g.35817247G>A | AA | 19 | 2.11 ± 0.81 | 0.778 | 31 | 2.06 ± 0.85 | 0.360 |
|  | GA | 146 | 2.25 ± 1.05 |  | 166 | 1.97 ± 0.82 |  |
|  | GG | 372 | 2.20 ±1.06 |  | 223 | 1.88 ± 0.82 |  |
|  | P value |  | |  |  | |  |
| g.35835329G>A | AA | 86 | 2.23 ± 1.04 | 0.973 | 70 | 2.07 ± 0.82 | 0.308 |
|  | GA | 249 | 2.20 ± 1.08 |  | 222 | 1.91 ± 0.82 |  |
|  | GG | 202 | 2.20 ± 1.02 |  | 128 | 1.91 ± 0.83 |  |
|  | P value |  | |  |  | |  |
| g.35835474G>T | GG | 465 | 2.22 ± 1.04 | 0.618 | 247 | 1.89 ± 0.83 | 0.505 |
|  | GT | 70 | 2.19 ±1.11 |  | 154 | 1.99 ± 0.82 |  |
|  | TT | 2 | 1.5 0.70 |  | 19 | 1.95 ± 0.78 |  |
| g.35841608T>C | CC | 3 | 1.67 ± 0.57 | 0.599 | 18 | 1.94 ± 0.80 | 0.504 |
|  | CT | 86 | 2.16 ± 1.05 |  | 155 | 1.99 ± 0.82 |  |
|  | TT | 448 | 2.22 ± 1.05 |  | TT | 1.89 ± 0.83 |  |
| g.35845633T>C | CC | 34 | 2.41 ± 1.13 | 0.156 | 6 | 1.50 ± 0.55 | 0.404 |
|  | CT | 187 | 2.10 ± 1.07 |  | 113 | 1.91 ± 0.84 |  |
|  | TT | 316 | 2.25 ± 1.03 |  | 300 | 1.95 ± 0.82 |  |
| g.35847837A>G | AA | 439 | 2.21 ± 1.05 | 0.659 | 236 | 1.89 ± 0.83 | 0.535 |
|  | AG | 94 | 2.19 ± 1.05 |  | 161 | 1.99 ± 0.83 |  |
|  | GG | 3 | 1.67 ± 0.58 |  | 23 | 1.96 ± 0.77 |  |
| g.35847864A>T | AA | 184 | 2.31 ± 1.04 | 0.17 | 130 | 2.04 ± 0.79 | 0.13 |
|  | TA | 269 | 2.18 ± 1.09 |  | 227 | 1.92 ± 0.82 |  |
|  | TT | 83 | 2.06 ± 0.90 |  | 62 | 1.79 ± 0.87 |  |
| g.35848079G>A | AA | 74 | 2.24 ± 1.00 | 0.865 | 65 | 2.11 ± 0.81 | 0.175 |
|  | GA | 256 | 2.18 ± 1.06 |  | 219 | 1.91 ± 0.82 |  |
|  | GG | 207 | 2.22 ± 1.06 |  | 136 | 1.89 ± 0.83 |  |
| g.35848108A>G | AA | 179 | 2.30 ± 1.04 | 0.296 | 114 | 2.03 ± 0.80 | 0.187 |
|  | GA | 273 | 2.18 ± 1.10 |  | 237 | 1.92 ± 0.81 |  |
|  | GG | 85 | 2.11 ± 0.89 |  | 69 | 1.80 ± 0.88 |  |
| g.35847912C>T | CC | 188 | 2.22 ± 1.03 | 0.968 | 129 | 1.91 ± 0.83 | 0.305 |
|  | CT | 260 | 2.21 ± 1.07 |  | 222 | 1.90 ± 0.82 |  |
|  | TT | 87 | 2.18 ± 1.04 |  | 69 | 2.07 ± 0.83 |  |
| g.35851829T>C | CC | 26 | 2.46 ± 1.14 | 0.231 | 12 | 1.96 ± 0.67 | 0.703 |
|  | CT | 185 | 2.12 ± 1.03 |  | 128 | 1.98 ± 0.85 |  |
|  | TT | 326 | 2.24 ± 1.05 |  | 280 | 1.91 ± 0.82 |  |
| g.35853589T>C | CC | 64 | 2.11 ± 0.96 | 0.719 | 71 | 2.08 ± 0.81 | 0.138 |
|  | CT | 245 | 2.22 ± 1.06 |  | 213 | 1.94 ± 0.83 |  |
|  | TT | 226 | 2.22 ± 1.06 |  | 135 | 1.84 ± 0.82 |  |
| g.35853637T>G | GG | 51 | 2.14±1.02 | 0.841 | 49 | 2.00 ± 0.79 | 0.508 |
|  | GT | 222 | 2.20±1.10 |  | 210 | 1.96 ± 0.83 |  |
|  | TT | 263 | 2.23±1.02 |  | 161 | 1.88 ± 0.83 |  |
| g.35853852T>C | CC | 29 | 2.24 ± 1.15 | 0.625 | 7 | 1.57 ± 0.53 | 0.352 |
|  | CT | 174 | 2.14 ± 1.05 |  | 96 | 2.00 ± 0.83 |  |
|  | TT | 333 | 2.24 ± 1.04 |  | 316 | 1.92 ± 0.82 |  |
| g.35867028T>C | CC | 95 | 2.14 ± 0.96 | 0.591 | - | - | - |
|  | CT | 258 | 2.20 ± 1.07 |  | - | - |  |
|  | TT | 184 | 2.27 ± 1.06 |  | - | - |  |
| g.105276945C>T | CC | 242 | 2.20 ± 1.07 | 0.697 | 284 | 1.88 ± 0.80 | 0.205 |
|  | CT | 223 | 2.19 ± 1.03 |  | 128 | 2.04 ± 0.86 |  |
|  | TT | 72 | 2.32 ± 1.02 |  | 7 | 2.00 ± 0.82 |  |
| g.105288550C>G | CC | 132 | 2.28 ± 1.00 | 0.663 | 133 | 1.89 ± 0.79 | 0.569 |
|  | GC | 237 | 2.20 ±1.05 |  | 184 | 1.98 ± 0.81 |  |
|  | GG | 167 | 2.17 ± 1.09 |  | 101 | 1.90 ± 0.89 |  |

1 ‘-’ denotes value is not available.
